# Closing the Diagnostic Gap in Early-Onset Colorectal Cancer: Red-Flag Symptoms, Screening, and Inherited Risk

**DOI:** 10.3390/cancers18172877

**Published:** 2026-09-05

**Authors:** Jose M. Martin-Moreno, Luis Cabañas-Alite, Ines E. Fernández-Benet, Manuel Sánchez-Casalongue, Antoni Alegre-Martinez

**Affiliations:** 1 Biomedical Research Institute—INCLIVA, University of Valencia, 46010 Valencia, Spain; jose.martin-moreno@uv.es (J.M.M.-M.); lcabanas@incliva.es (L.C.-A.); ifernandez@incliva.es (I.E.F.-B.); masanchez@incliva.es (M.S.-C.); 2 Department of Preventive Medicine, Medical School, University of Valencia, 46010 Valencia, Spain; 3 Faculty of Health Sciences, Miguel de Cervantes European University, C. del Padre Julio Chevalier, 2, 47012 Valladolid, Spain

**Keywords:** early-onset colorectal cancer, young-onset colorectal cancer, colorectal cancer screening, diagnostic delay, red-flag symptoms, family history, germline pathogenic variants, Lynch syndrome, polygenic risk scores, risk stratification

## Abstract

**Background/Objectives**: Early-onset colorectal cancer (EOCRC), diagnosed before age 50 years, is increasing in many populations and usually arises outside routine screening pathways. We synthesized clinical, demographic, familial, and genetic evidence relevant to risk-stratified early detection. **Methods**: Two core PubMed searches covering publications from 1 January 2010 through 1 June 2026 were supplemented by targeted PubMed searches and reference-list screening. The present review cited 82 journal publications and synthesized the findings qualitatively; no formal risk-of-bias assessment, certainty-of-evidence grading, or de novo quantitative pooling was performed. **Results**: EOCRC commonly involves the distal colon or rectum and presents with hematochezia, abdominal pain, altered bowel habits, or iron-deficiency anemia, with diagnostic intervals of several months. Screening colonoscopy in average-risk adults aged 45–49 years has a clinically relevant yield, although generally lower than in older adults, and direct evidence of reduced incidence or mortality remains limited. Risk rises with age before 50; sex and race or ethnicity provide limited, context-dependent discrimination. Family history is a consistent marker, although most cases lack documented familial aggregation. Pathogenic germline variants explain a minority, and polygenic risk scores are not established for routine practice. **Conclusions**: Early detection should combine timely investigation of warning signs, assessment of family history, equitable access to diagnostics, germline testing for patients with EOCRC, cascade testing, and prospective validation of population-specific models.

## Simple Summary

Early-onset colorectal cancer is diagnosed before the age of 50, and its incidence is rising in many populations. Because most younger adults are not routinely offered screening, diagnosis usually follows the onset of symptoms. Rectal bleeding, persistent abdominal pain, sustained changes in bowel habits, and unexplained iron-deficiency anemia warrant timely evaluation. A family history of colorectal cancer or advanced colorectal polyps, or an inherited cancer-predisposition syndrome, may identify people who require earlier or more intensive surveillance. Screening beginning at age 45 can detect clinically important precancerous lesions, although direct evidence that starting population screening before age 50 reduces colorectal cancer incidence or mortality remains limited. A pragmatic strategy should combine prompt investigation of warning signs, systematic collection of family history, appropriate genetic counseling and testing, and equitable access to high-quality diagnostic and screening services.

## 1. Introduction

Early-onset colorectal cancer (EOCRC), defined as colorectal cancer diagnosed before age 50 years, is increasing in multiple countries. In contrast, incidence in older adults has stabilized or declined in settings with organized screening [1,2]. In this review, EOCRC was operationally defined as CRC diagnosed before age 50, consistent with the age threshold most commonly used in the EOCRC literature. However, definitions have varied across individual studies, with some using alternative age cut-offs such as <45 or <55 years. Accordingly, the studies summarized in this review were not required to use an identical age cut-off; their original definitions were retained when reporting individual findings. This variation should be considered when comparing effect estimates across studies. Interpretation of EOCRC risk factors also requires consideration of changes in screening recommendations over the study period. In 2018, the American Cancer Society recommended initiation of average-risk CRC screening at age 45 years, followed by the U.S. Preventive Services Task Force in May 2021. These changes may have influenced the detection of CRC among individuals aged 45–49 years in more recent studies, potentially increasing identification of otherwise asymptomatic disease compared with earlier study periods. However, screening recommendations do not alter the epidemiological definition of EOCRC used in this review; rather, they may affect case ascertainment and the observed distribution of risk factors and clinical presentation among younger adults. Although relative increases are substantial, absolute EOCRC incidence remains much lower than in older adults, an important consideration for population-wide screening policies [3]. The burden is uneven across populations and regions, with marked racial/ethnic and socioeconomic disparities in age at diagnosis and outcomes [4,5,6,7,8].

Recent international evidence places EOCRC within a broader increase in cancers diagnosed among younger adults, while also revealing a particularly distinctive age pattern for colorectal cancer. Across 42 countries with high-quality registry data, colorectal cancer incidence increased among adults aged 20–49 years in 88% of countries, with a median annual increase of 1.45%, compared with 0.37% among adults aged ≥50 years; trends were more unfavorable in younger adults in 69% of countries [9]. This divergence may partly reflect the preventive effects of organized screening and premalignant-lesion removal in older populations. Still, it has also prompted consideration of generation-specific mechanisms, including cumulative environmental and behavioral exposures beginning early in life, alterations in the gut microbiome and exposome, and age-related molecular, mutational, and immunological differences [10]. Complementing these epidemiological observations, prospective UK and US data suggest that more recent birth cohorts exhibit greater acceleration in systemic biological aging, which has been associated with early-onset gastrointestinal and colorectal cancers. At the same time, proteomics-based analyses have linked accelerated adipose tissue aging with EOCRC [11]. Although these emerging findings remain investigational and do not establish causality, they reinforce the view that chronological age alone may not adequately capture EOCRC susceptibility. At the health-system level, screening and referral pathways remain largely designed around older populations, supporting precision-based approaches that integrate clinical warning signs, family history, and inherited susceptibility to identify younger adults most likely to benefit from timely diagnostic evaluation [12].

Against this background, EOCRC poses a particular diagnostic challenge because it is frequently detected after symptom onset rather than through screening. Rectal bleeding or hematochezia, abdominal pain, altered bowel habits, diarrhea, and iron-deficiency anemia are frequent presenting features, and mean or median diagnostic intervals of approximately four to six months have been reported [2,7,13,14]. Attribution to benign conditions, repeated healthcare encounters, and delayed colonoscopy are frequently described, although observational evidence does not establish that these factors account for all diagnostic delays.

EOCRC is also more frequently located in the distal colon and rectum than late-onset colorectal cancer. At the same time, its frequent presentation outside routine screening pathways highlights the limitations of age-based screening thresholds [2,15,16,17,18]. Familial aggregation and inherited susceptibility provide complementary opportunities for earlier risk identification. A family history of colorectal cancer is consistently associated with increased EOCRC risk, while a history of advanced colorectal polyps in first-degree relatives may further refine familial risk assessment [17,19]. Pathogenic germline variants in DNA mismatch repair genes, polyposis-associated genes, and other cancer-predisposition genes account for a clinically relevant subset of EOCRC, including some cases without an obvious hereditary syndrome [20,21]. Polygenic risk scores may further stratify susceptibility, although their clinical implementation and added value beyond family history and established risk markers remain to be validated [22,23]. Together, these markers may help identify younger individuals who warrant structured familial risk assessment and referral for germline genetic evaluation. This narrative review synthesizes evidence on clinical presentation, diagnostic pathways, demographic and familial markers, and inherited susceptibility to inform risk-stratified detection and prevention of EOCRC.

Although previous reviews have addressed important components of EOCRC, including epidemiology, screening, hereditary susceptibility, and diagnostic delay, these domains have often been considered separately or with a primary emphasis on a single aspect. The distinctive contribution of the present review is its clinically oriented integration of symptom-triggered diagnostic escalation, screening evidence, familial and inherited susceptibility, and implementation and equity considerations within a single risk-stratified early-detection framework. Rather than proposing a new quantitative risk model, the review synthesizes these complementary evidence streams to identify practical points at which earlier recognition, diagnostic evaluation, genetic assessment, and targeted surveillance may be linked in younger adults.

### Objectives

- To summarize and critically appraise evidence from meta-analyses and large observational studies on selected clinical, demographic, familial, genetic, and detection-related determinants for EOCRC;
- To examine, where comparative evidence was available, whether selected clinical, demographic, familial, genetic, and detection-related determinants differed between EOCRC and late-onset CRC;
- To translate the available evidence into clinically actionable pathways for symptom-based diagnostic escalation, familial/genetic assessment, and risk-adapted early detection.

This study was conducted as part of Work Package 2 of the ONCODIR project (HORIZON-MISS-2022-CANCER-01), which aims to advance evidence-based approaches for colorectal cancer prevention in Europe.

## 2. Materials and Methods

### 2.1. Study Design and Scope

This narrative review examined clinical, anatomical, demographic, familial, and inherited-susceptibility markers relevant to the earlier detection and risk stratification of EOCRC, generally defined as colorectal cancer diagnosed before age 50 years. It was developed within the ONCODIR Work Package 2 framework (HORIZON-MISS-2022-CANCER-01) and forms one of two companion narrative reviews derived from a shared literature-identification process. The present review focused on tumor location; screening and diagnostic pathways; prediagnostic symptoms and diagnostic intervals; age, sex, and race or ethnicity; family history of colorectal cancer or advanced colorectal polyps; pathogenic germline variants and hereditary cancer syndromes; and polygenic susceptibility. Modifiable lifestyle, metabolic, inflammatory, pharmacological, dietary, and socioeconomic determinants were addressed separately in the companion review on life-course determinants and primary prevention [24].

### 2.2. Literature Search

Two complementary core PubMed searches were performed to identify meta-analyses and primary observational studies, including cohort, case–control, prospective, and population-based designs. Publications dated from 1 January 2010 through 1 June 2026 were considered in the core searches.

To identify meta-analyses, we used the following PubMed strategy: (“colorectal cancer”[Title/Abstract] OR “colon cancer”[Title/Abstract] OR “rectal cancer”[Title/Abstract]) AND (“early-onset”[Title/Abstract] OR “young adults”[Title/Abstract] OR “under 50”[Title/Abstract] OR “younger than 50”[Title/Abstract]) AND (“risk factor”[Title/Abstract] OR “risk factors”[Title/Abstract] OR “etiology”[Title/Abstract] OR “determinant”[Title/Abstract] OR “associated with”[Title/Abstract]) AND (Meta-Analysis[ptyp]) AND (“1 January 2010”[Date—Publication]: “1 June 2026”[Date—Publication])

To identify primary observational studies, we used: (“colorectal cancer”[Title/Abstract] OR “colon cancer”[Title/Abstract] OR “rectal cancer”[Title/Abstract]) AND (“early-onset”[Title/Abstract] OR “young adults”[Title/Abstract] OR “under 50”[Title/Abstract] OR “younger than 50”[Title/Abstract]) AND (“risk factor”[Title/Abstract] OR “risk factors”[Title/Abstract] OR “etiology”[Title/Abstract] OR “associated with”[Title/Abstract]) AND (cohort[Title/Abstract] OR “case-control”[Title/Abstract] OR “prospective”[Title/Abstract] OR “population-based”[Title/Abstract]) AND (“1 January 2010”[Date—Publication]: “1 June 2026”[Date—Publication])

Both core PubMed searches were conducted on 2 June 2026, and no language restrictions were applied. Articles published after this date were not included in the formal search results or evidence synthesis; a limited number of subsequently published publications were cited solely as contextual sources to provide updated background information. Because the core search syntax was centered on broad risk-factor terminology, the searches were supplemented, where necessary, by domain-specific PubMed searches and reference-list screening of key reviews and relevant primary studies. The supplementary searches focused on evidence domains that were not expected to be fully captured by the broad risk-factor terminology of the core strategies, including clinical presentation and red-flag symptoms, diagnostic intervals and healthcare encounters, screening and endoscopic yield, tumor location, age, sex and race or ethnicity, family history and familial colorectal polyps, pathogenic germline variants and hereditary cancer syndromes, and polygenic susceptibility.

For the supplementary searches, combinations of terms related to early-onset or young-onset colorectal cancer were combined with domain-specific terms corresponding to these topics, including terms related to symptoms, screening and endoscopy, tumor location or subsite, demographic characteristics, family history or colorectal polyps, germline pathogenic variants and hereditary cancer syndromes, and polygenic risk. Reference lists of key reviews and relevant primary studies were additionally screened to identify publications addressing these domains that were not captured by the core or targeted PubMed searches.

Publications identified through the supplementary procedures were considered relevant when they addressed EOCRC, young-onset colorectal neoplasia, or advanced colorectal neoplasia in younger adults and provided evidence relevant to tumor location, screening or diagnostic pathways, prediagnostic symptoms or diagnostic intervals, demographic markers, family history or familial polyps, pathogenic germline variants, hereditary cancer syndromes, or polygenic susceptibility. Publications were not retained when they fell outside the scope of the review or did not provide evidence relevant to early detection, diagnostic pathways, familial or inherited susceptibility, or risk stratification.

The supplementary searches and reference-list screening were performed iteratively, consistent with the narrative-review design. These procedures were not prospectively logged as separate search streams; therefore, their individual numerical contributions cannot be reliably reconstructed from the available records. We therefore report the overall core-search screening and final publication allocation rather than assigning retrospective numbers to individual supplementary procedures. Additional contextual publications released after the core search cutoff were identified during manuscript preparation and were used to frame the Introduction and Discussion.

### 2.3. Relevance Assessment and Thematic Allocation

Records retrieved from the two core PubMed searches were merged and deduplicated. Titles and abstracts were screened for relevance to either of the two companion review domains, and full texts were consulted as needed to assess thematic relevance and interpret study findings. Publications were allocated to (i) the life-course determinants and primary-prevention review, (ii) the clinical presentation, diagnostic pathways, and inherited-susceptibility review, or (iii) both reviews, according to the evidence contributed to each research question.

The two core searches identified 362 records, comprising 23 records from the meta-analysis search and 339 from the observational-study search. After removal of seven duplicates, 355 unique records were screened by title and abstract. The final citation set assembled through core screening and supplementary literature identification comprised 114 unique journal publications across the two companion reviews. Of these, 43 were cited in both reviews, 32 only in the life-course determinants and primary-prevention review, and 39 only in the present review, which addresses clinical presentation, diagnostic pathways, and inherited susceptibility. Accordingly, the present review cited 82 journal publications.

Publications were considered eligible for the present review when they addressed EOCRC, young-onset colorectal neoplasia, or advanced colorectal neoplasia in younger adults and contributed evidence on tumor location, screening or diagnostic pathways, prediagnostic symptoms, diagnostic intervals, demographic markers, family history or familial colorectal polyps, pathogenic germline variants, hereditary cancer syndromes, or polygenic susceptibility. Studies were excluded if they did not address the review population or scope, did not provide information relevant to these domains, or did not contribute evidence on early detection, diagnostic pathways, familial or inherited susceptibility, or risk stratification.

The core literature-identification process and final publication allocation are summarized in Supplementary Figure S1: PRISMA-style flow diagram of literature identification, screening, and inclusion across the two companion narrative reviews. The complete cross-reference and broad thematic classification of the cited journal publications are provided in Supplementary Table S1.

Publications relevant to the present review addressed EOCRC or young-onset colorectal neoplasia. They reported evidence on tumor location, screening or diagnostic pathways, prediagnostic symptoms, diagnostic intervals, demographic markers, family history or familial colorectal polyps, pathogenic germline variants, hereditary cancer syndromes, or polygenic susceptibility. Studies of advanced colorectal neoplasia in younger adults were considered when they informed screening yield, early detection, or familial risk assessment.

### 2.4. Evidence Prioritization and Narrative Synthesis

Evidence was organized qualitatively by domain. Greater weight was given to meta-analyses, population-based cohorts, prospective studies, large case–control studies, and studies directly comparing EOCRC with later-onset colorectal cancer. When several publications addressed the same domain, greater weight was given to pooled estimates, larger sample sizes, population-based designs, adjustment for major confounders, and direct relevance to EOCRC detection or inherited susceptibility. Retrospective findings, single-center molecular studies, preprints, and isolated extreme estimates were interpreted cautiously. For synthesis, evidence was also interpreted according to its primary role in the EOCRC pathway, distinguishing factors associated with disease development or inherited susceptibility, markers relevant to prediagnostic detection, and features related to diagnostic pathways or delay. These categories were not mutually exclusive, as some markers may contribute to more than one stage of the pathway. No formal risk-of-bias assessment, certainty-of-evidence grading, or de novo quantitative pooling was performed.

## 3. Results

**Figure 1:**
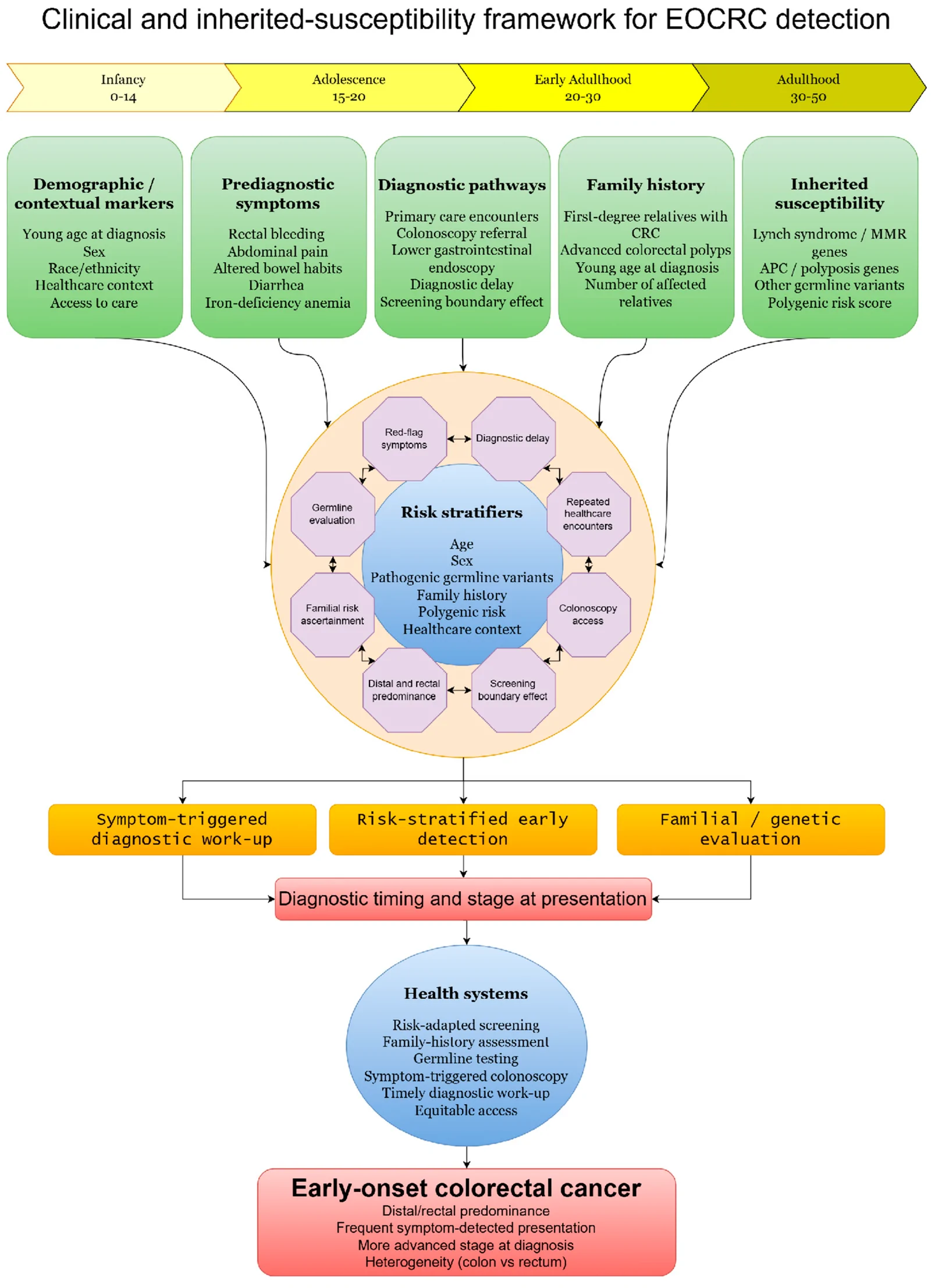
Clinical and inherited-susceptibility framework for risk-stratified detection of early-onset colorectal cancer. The framework illustrates how demographic and healthcare-contextual markers, prediagnostic symptoms, diagnostic pathways, family history, and inherited susceptibility may converge to guide symptom-triggered diagnostic work-up, familial/genetic evaluation, and risk-adapted early detection.

The present review drew on 82 journal publications addressing EOCRC epidemiology, clinical presentation, diagnostic pathways, tumor subsite, demographic markers, family history, inherited susceptibility, surveillance, and health-system determinants. Of these, 43 were also cited in the companion review, and 39 were specific to the present review. Figure 1 summarizes the clinical and inherited-susceptibility framework, while factor-specific evidence is presented in Table 1 and synthesized by domain below.

### 3.1. Anatomical Distribution and Clinical Phenotype

EOCRC is more frequently diagnosed in the distal colon and rectum than late-onset colorectal cancer, with distal tumor location associated with diagnosis before age 50 years (OR 1.66) [25]. This anatomical pattern is a clinically relevant feature of the presentation of EOCRC and may contribute to its symptom profile and diagnostic trajectory. Although the mechanisms underlying the distal/rectal predominance remain incompletely understood, the consistency of this pattern across cohorts supports considering tumor subsite when interpreting EOCRC heterogeneity and designing risk-stratified detection strategies. Several cohorts have also reported a higher proportion of advanced-stage disease at diagnosis among younger patients. However, the magnitude and consistency of this pattern vary across populations and healthcare contexts [16,18,44].

### 3.2. Screening and Diagnostic Pathways

Evidence indicates a clinically relevant detection yield for screening before age 50, although uptake remains limited. Evidence on screening before age 50 is largely derived from studies of colonoscopy yield rather than from direct comparisons between screened and unscreened populations. In a systematic review and meta-analysis of asymptomatic average-risk individuals younger than 50 years undergoing screening colonoscopy, the pooled prevalence of advanced colorectal neoplasia was 2.2%. Among individuals aged 45–49 years, advanced neoplasia was detected in 3.6%, indicating a clinically relevant yield, although somewhat lower than the 4.2% observed among individuals aged 50–59 years [26]. These findings support the potential value of screening starting at age 45 years, which may yield clinically meaningful results, although they do not directly establish reductions in EOCRC incidence or mortality. Evidence on lower gastrointestinal endoscopy before age 50 remains limited and inconsistent, and observational associations are susceptible to confounding by indication [45]. By contrast, measures of prior screening history may be confounded by selective screening of higher-risk individuals, yielding null associations in some observational analyses [46]. Population-based data indicate that outpatient visits increase mainly during the year preceding young-onset colorectal cancer diagnosis, when colorectal cancer-related or red-flag presentations become more frequent. This pattern suggests a narrow opportunity for diagnostic escalation rather than evidence that greater primary care utilization reduces EOCRC risk [27]. Diagnostic intervals of approximately four to six months are commonly reported in symptomatic younger adults [13,28]. Hematochezia or rectal bleeding, abdominal pain, and altered bowel habits are frequent presenting features. Attribution to benign conditions and repeated healthcare encounters before colonoscopy have also been described. Still, the available observational evidence does not establish that they cause all diagnostic delays [18,28].

### 3.3. Prediagnostic Symptoms and Diagnostic Escalation

Prediagnostic gastrointestinal symptoms and anemia-related findings consistently distinguish EOCRC cases from controls. Abdominal pain, rectal pain, rectal bleeding, hematochezia, and rectal tenesmus have shown strong associations with EOCRC, although some symptoms do not clearly differentiate EOCRC from later-onset CRC [47,48]. Anemia, particularly iron-deficiency anemia, gastrointestinal bleeding, and anal or rectal conditions are recurrent predictors of EOCRC. They are frequently documented before diagnosis, supporting timely diagnostic evaluation when these findings persist or remain unexplained [18,29]. Prediction models have also incorporated healthcare-utilization markers, such as prior endoscopy and CT encounters, although their clinical utility for EOCRC risk stratification remains to be validated [18,29]. Low serum ferritin may represent an additional diagnostic clue in younger adults with suspected colorectal pathology. However, its role in selecting individuals for endoscopic evaluation remains unvalidated and is currently supported only by retrospective evidence [30].

### 3.4. Demographic and Contextual Markers

Within adults aged <50 years, EOCRC risk increases progressively with age. Multiple studies reported higher odds of EOCRC or advanced colorectal neoplasia with each additional year of age, with estimated increases of approximately 5–8% per year [18,31,32,33,49,50]. Risk rises steadily from early adulthood to the late 40s, supporting age-stratified risk assessment and heightened vigilance as individuals approach conventional screening ages [18,31,32,33,49]. Male sex is associated with a modestly increased risk of EOCRC or advanced early-onset neoplasia in most studies, with pooled estimates suggesting approximately 18% higher risk in men [34,35,51]. Similar associations have been reported across several observational cohorts [31,32,36,49,50,52,53]. However, findings are inconsistent, with some studies reporting no association and others suggesting lower EOCRC odds among men [25,46,54,55]. Overall, sex appears to be a weak-to-moderate risk factor and is most informative when incorporated into multivariable risk-stratification models rather than used independently [25,34,35,36,46,50,51,52,53,54,55]. Associations between ethnicity/race and EOCRC are inconsistent across populations. Some pooled analyses reported higher EOCRC risk among White/Caucasian individuals compared with other ethnic groups [34]. In contrast, others identified increased risk or burden among non-Hispanic Black, non-Hispanic Asian, African American, or specific regional populations [37,47,51]. Hispanic/Latino ethnicity has been associated with lower risk in some studies [52], while others have found no independent association after adjustment [31,32]. Racial and ethnic differences may persist after accounting for metabolic and lifestyle factors in some analyses [38], suggesting contributions from population structure, environmental exposures, healthcare access, and diagnostic pathways. Overall, race and ethnicity may provide contextual information relevant to EOCRC risk stratification. Still, associations remain heterogeneous and context-dependent and should not be interpreted as deterministic measures of biological susceptibility [31,32,34,37,38,47,51,52].

### 3.5. Family History and Familial Polyps

Family history of colorectal cancer is among the strongest and most consistent EOCRC risk factors. Meta-analyses indicate that a family history of CRC approximately doubles the risk of EOCRC, with similar estimates reported across large observational studies [17,34,35,36,39,56]. Risk increases when affected relatives are diagnosed at younger ages, when multiple first-degree relatives are affected, and, in some studies, when the affected relative is a sibling or when colon rather than rectal cancer is considered [39,40,45]. Family history is also associated with advanced colorectal neoplasia before age 50 [32,53]. In contrast, a family history of advanced colorectal polyps, particularly advanced adenomas, multiple lesions, or polyps diagnosed before age 50, confers increased CRC risk with a dose–response relationship according to the number of affected relatives [19].

Although EOCRC patients report family history more frequently than late-onset CRC patients [37,57,58], most cases occur without a documented family history of CRC [59,60]. In one prospective cohort, family history of non-CRC cancers alone was not independently associated with EOCRC risk; this finding requires replication in other populations [54]. Hereditary predisposition contributes substantially but does not account for most EOCRC, as only a minority of patients carry pathogenic germline variants in cancer susceptibility genes [51]. These findings support systematic assessment of family history, including degree of relationship, age at diagnosis, number of affected relatives, and history of advanced polyps, to guide risk-adapted screening and genetic evaluation.

### 3.6. Inherited Susceptibility and Polygenic Risk

Pathogenic germline variants contribute substantially to EOCRC risk, although most cases remain unexplained by recognized hereditary syndromes. Pathogenic germline variants are enriched in familial EOCRC but are also detected in apparently sporadic cases, involving DNA mismatch repair genes, polyposis-associated genes, and other cancer-predisposition genes [20,21]. In a meta-analysis of Lynch syndrome/HNPCC surgical cohorts, the mean age at CRC onset was approximately 45.5 years, and metachronous CRC occurred in 22.7% of patients treated with segmental colectomy compared with 4.7% after extensive colectomy [61]. Additional pathogenic variants have been identified in BMPR1A, MUTYH, POLD1, TP53, and other cancer predisposition genes [21]. EOCRC patients also more frequently develop Lynch-related malignancies [37], although some early-onset MSI-H tumors with MLH1 methylation appear biologically distinct from classical Lynch syndrome and lack germline MLH1 mutations [62]. Beyond canonical CRC genes, pathogenic variants in DNA repair, checkpoint, and other tumorigenesis-related pathways may contribute to EOCRC susceptibility [20]. Rare variants in genes such as MCM8, LRP6, and PTPN12 have been implicated in Lynch-like or familial EOCRC, although evidence remains limited and interpretation is often complicated by variants of uncertain significance [41,63]. Clinically occult hereditary predisposition may also occur, as pathogenic TP53 variants have been identified in EOCRC patients lacking classical hereditary cancer syndrome criteria [42].

Polygenic susceptibility may contribute to the risk of EOCRC. Polygenic risk scores (PRS) based on common CRC-associated variants have been associated with EOCRC risk in several studies, with the magnitude of the association varying by study population and analytical approach. Archambault et al. reported 3.73-fold higher odds of EOCRC in the highest versus lowest PRS quartile, which increased to 4.26-fold among those without a first-degree family history of CRC [22]. X. Chen et al. similarly found an independent association between higher PRS and EOCRC risk, although the interaction between PRS and age was not statistically significant [43]. Some evidence therefore suggests stronger relative associations for EOCRC than for later-onset CRC [22,43]. However, the clinical utility of PRS for routine EOCRC risk assessment remains unestablished. Genetic loci associated with EOCRC are linked to pathways involved in Wnt signaling, cell-cycle regulation, DNA repair, and immune function [17,64]. However, evidence for individual SNPs remains inconsistent, with only a limited number showing reproducible EOCRC-specific associations [17,65,66]. These discrepancies highlight the need for standardized measurement and validation in larger cohorts. Overall, germline testing should be offered to all patients with EOCRC, ideally before surgery and irrespective of family history, in accordance with the international DIRECt consensus [67]. Polygenic risk scores may eventually complement germline testing and family-history assessment. Still, their clinical implementation remains investigational and requires prospective validation, including evaluation of population transferability, calibration, and added value beyond established risk markers [20,22,43,46].

## 4. Discussion

EOCRC prevention and earlier detection cannot be based on a single age threshold alone. The most actionable opportunities currently lie in prompt diagnostic escalation for symptomatic younger adults, systematic assessment of familial and inherited risk, and equitable implementation of risk-stratified early-detection pathways. A key contribution of this review is to connect these domains rather than treating screening, symptom-based diagnosis, familial risk, and inherited susceptibility as separate strategies, thereby framing EOCRC early detection as a continuum spanning clinical presentation, risk assessment, diagnostic evaluation, genetic assessment, and surveillance.

### 4.1. Screening, Symptom-Triggered Diagnosis, and Risk-Stratified Prevention

Historically, average-risk CRC screening was initiated at age 50. Still, the increasing incidence of EOCRC and its frequent presentation outside organized screening pathways expose the limitations of age-based eligibility alone [6,8,16,44]. In settings where screening begins at age 50, the abrupt increase in diagnoses, early-stage detection, and survival around this threshold suggests a potential screening-boundary effect, whereby preclinical cancers remain undetected before conventional eligibility [68]. Importantly, the markers considered in this review do not occupy the same position in the clinical pathway. Familial, demographic, and inherited markers primarily inform susceptibility and risk stratification. In contrast, red-flag symptoms and anemia-related findings are mainly relevant to prediagnostic detection, and healthcare encounters or attribution to benign conditions relate more directly to diagnostic pathways and potential delay.

The divergence in colorectal cancer incidence between younger and older adults should not be interpreted as evidence of a single age-specific causal mechanism. It may instead reflect the combined influence of changing life-course exposures in recent generations and the preventive effect of organized screening in older populations, in whom colonoscopy and removal of premalignant lesions may attenuate incidence trends. Accordingly, comparisons between age groups should recognize that most younger adults remain outside preventive screening pathways, whereas screening-eligible populations may have benefited from decades of secondary prevention. These findings should prompt action rather than alarm: priorities include improved recognition of persistent warning symptoms, timely access to diagnostic colonoscopy, systematic assessment of familial and inherited susceptibility, and better-calibrated and equitable criteria for risk-adapted screening. At the same time, the substantially greater absolute burden of colorectal cancer among older adults must remain central to prevention and healthcare planning [2,69].

Earlier screening may reduce late-stage disease in selected populations, particularly where clinical suspicion remains low; however, the relatively low absolute incidence of EOCRC limits the efficiency of broad expansion of population-wide screening [69,70,71,72,73]. The near-term challenge is therefore not only determining the optimal screening age, but also reducing delays in diagnostic evaluation among symptomatic younger adults. EOCRC is predominantly symptom-detected, and diagnostic intervals of several months are frequently reported. Nonspecific or intermittent symptoms, low clinical suspicion, and repeated healthcare encounters before colonoscopy may contribute in some cases, although their causal role cannot be established from the available observational evidence. Recognition of symptom clusters, iron-deficiency anemia, and recurrent presentations may facilitate earlier escalation of diagnosis in primary care [14]. The effectiveness of any screening-age reduction will also depend on equitable implementation, uptake, and timely access to diagnostic colonoscopy and downstream care [2]. Risk-stratified approaches may offer an alternative or complement to universal age-based strategies. In younger adults, potentially useful inputs include age in the <50-year range, red-flag symptoms, anemia-related findings, prior healthcare encounters, family history of CRC or advanced colorectal polyps, and validated inherited risk markers. Family history assessment may be strengthened by recording the degree of relationship, age at diagnosis, number of affected relatives, and the presence of advanced- or early-onset colorectal polyps in relatives [19]. However, proposed decision-support tools require prospective validation, integration into electronic health records, clinician acceptance, and alignment with clinical guidelines before widespread implementation [74,75,76,77,78].

### 4.2. Hereditary Risk and Genetic Evaluation

EOCRC prevention is particularly challenging in hereditary and high-risk settings, where age-based thresholds and family history alone are insufficient. Because clinically occult hereditary predisposition cannot be reliably excluded by the absence of familial aggregation, the international DIRECt consensus recommends offering germline testing to all patients with EOCRC, ideally before surgery, to inform clinical management and familial surveillance [67]. The evidence underlying inherited-risk estimates remains heterogeneous and derives from observational cohorts, genetic studies, and populations with differing referral and testing practices; therefore, the strength and generalizability of individual risk estimates should be interpreted with caution. The recommendation for universal germline testing in EOCRC is based on the DIRECt consensus rather than on a de novo quantitative estimate from the present review. In high-penetrance syndromes such as Peutz–Jeghers syndrome, the optimal timing and intensity of surveillance remain uncertain, and current strategies may still fail to prevent or detect malignancy early in some young individuals [79]. EOCRC is enriched for pathogenic germline variants in DNA mismatch repair genes, polyposis-associated genes, and other cancer-predisposition genes. However, most cases remain clinically classified as sporadic and lack a recognized hereditary syndrome [16]. Family history, particularly sibling history and a family history of colorectal polyps, may exert stronger relative effects in younger-onset disease and in more recent birth cohorts [19,40]. Polygenic risk scores may eventually complement family history and monogenic testing, although their incremental clinical value, population transferability, calibration, and implementation requirements remain to be established [22,43]. Emerging somatic and transcriptomic signatures may eventually refine biological classification and EOCRC stratification; however, their clinical utility for prediagnostic risk assessment remains unproven.

**Figure 2:**
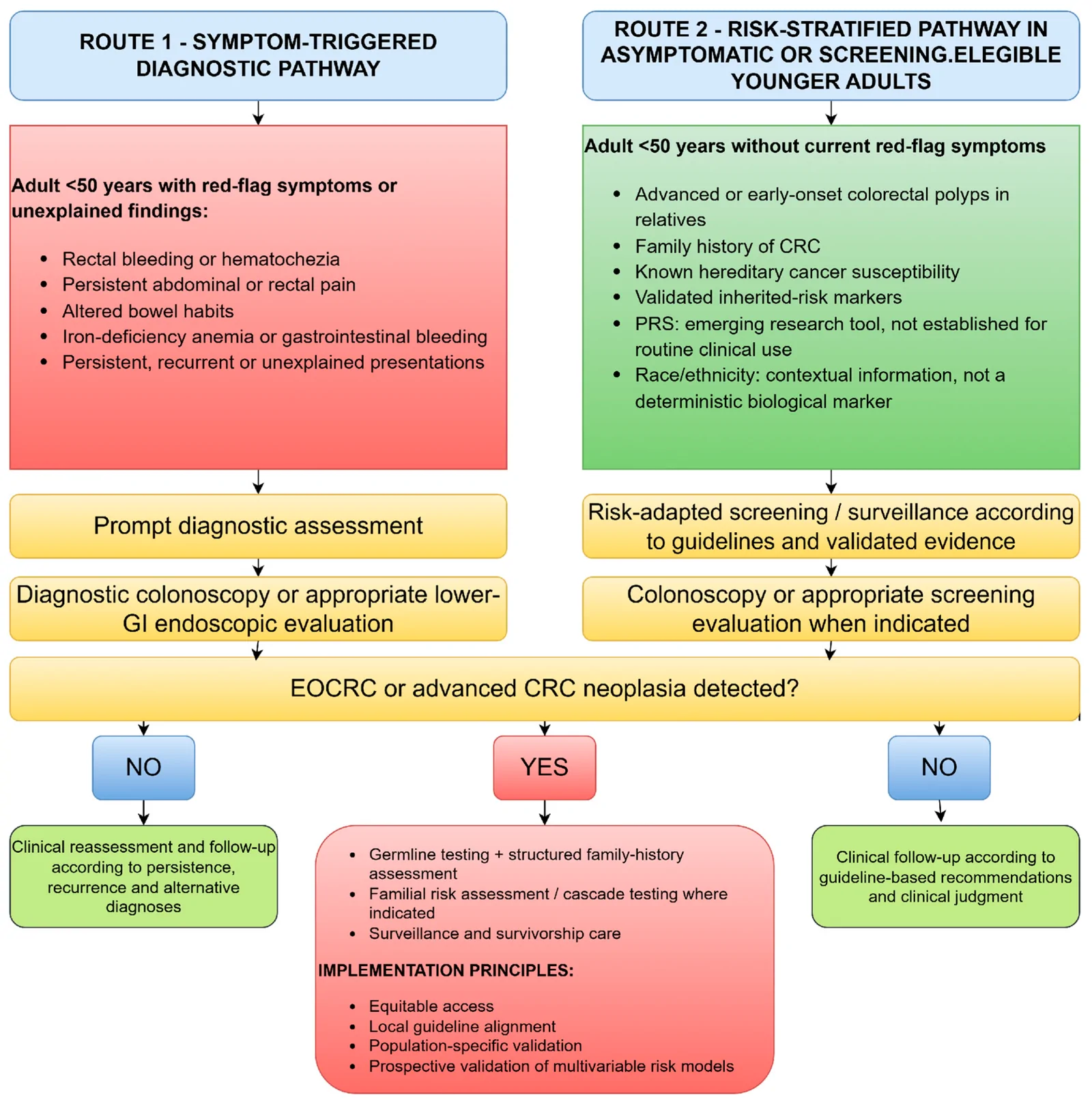
Proposed risk-stratified pathway for early detection, genetic assessment and surveillance in adults younger than 50 years.

Based on the evidence synthesized in this review, we propose a risk-stratified clinical pathway that integrates symptom assessment, individual risk estimation, diagnostic or screening colonoscopy, germline testing, and subsequent surveillance for adults younger than 50 years (Figure 2). The proposed framework is intended as a conceptual approach to support earlier recognition and individualized risk assessment rather than as a prescriptive clinical algorithm.

### 4.3. Surveillance After Lesions or Cancer

Risk reduction extends beyond initial detection. Evidence suggests that intensifying post-resection surveillance based solely on younger age at CRC onset may not be warranted, indicating that age alone is insufficient to justify shorter colonoscopy intervals [80]. Survivorship care may also require adaptation to age-specific risks of second primary malignancies, supporting EOCRC-aware long-term follow-up strategies [81]. Comparative survival estimates between early- and late-onset CRC have varied across settings, reinforcing the need for context-specific interpretation of age-related outcomes [74].

### 4.4. Equity and Implementation

Screening effectiveness depends not only on eligibility criteria but also on access, timeliness, diagnostic capacity, and the quality of downstream care. Persistent racial and ethnic disparities in EOCRC outcomes highlight the need for interventions addressing inequities in access to diagnostic evaluation, treatment delivery, and follow-up care [4,5]. These disparities should not be interpreted as evidence of race- or ethnicity-specific biological susceptibility alone, as observed differences may also reflect population structure, environmental exposures, healthcare access, socioeconomic conditions, and differences in diagnostic and treatment pathways. System-level gaps, including delayed treatment initiation and reduced treatment continuity in some younger populations, may further diminish the potential benefits of early detection [7,82]. These concerns also affect the implementation and transportability of risk-stratified approaches. Prediction models and genetic tools must be validated across diverse populations and healthcare settings rather than extrapolated from narrowly represented cohorts. This limitation is particularly relevant for low- and middle-income countries (LMICs), which appear to be underrepresented in the evidence base considered in this review. Differences in screening infrastructure, access to colonoscopy, availability of genetic testing, referral pathways, cancer registration, and healthcare resources may affect both the observed risk patterns and the feasibility of the proposed early-detection strategies. Accordingly, the findings should not be assumed to be directly transferable to LMIC settings without local validation and adaptation. More broadly, evidence gaps in guidelines derived predominantly from older populations limit their applicability to EOCRC and reinforce the need for age-relevant data on detection, surveillance, and management [83]. Within Europe, organized age-based colorectal cancer screening remains the foundation of population prevention, but EOCRC frequently arises below conventional eligibility thresholds. Rather than replacing established programs, the present evidence supports complementing them with risk-adapted pathways: systematic recording of family history, including advanced colorectal polyps, in primary care; timely referral for genetic assessment when indicated; and rapid diagnostic escalation for younger adults with red-flag symptoms or anemia-related findings. These measures are consistent with Europe’s Beating Cancer Plan and the 2022 Council Recommendation on cancer screening [84], while addressing persistent inequalities in access to early diagnosis and follow-up care across health systems [85,86]. Within the ONCODIR framework, this perspective may help translate clinical and epidemiological evidence into practical decision-support strategies linking risk assessment, early detection, and equitable implementation across European settings. Overall, the available evidence supports a tiered approach to EOCRC prevention and early detection: rapid diagnostic escalation for younger adults with red-flag symptoms or anemia-related findings; structured family-history assessment that includes advanced colorectal polyps; appropriate germline evaluation in affected patients; and prospective validation of multivariable risk models incorporating clinical and inherited markers. Given the low absolute incidence of EOCRC in many populations, targeted strategies may ultimately prove more efficient than universal lowering of screening thresholds, provided that they are implemented equitably and tested in real-world clinical settings.

### 4.5. Strengths and Limitations

This narrative review used two core PubMed strategies targeting meta-analyses and observational EOCRC studies published from 1 January 2010 through 1 June 2026, supplemented by targeted PubMed searches and reference-list screening. Its structured organization by clinically relevant domains—tumor location, screening, prediagnostic clinical features and diagnostic work-up, age, sex, race or ethnicity, family history, and inherited susceptibility—supports an integrated synthesis of factors relevant to early detection and risk stratification. The inclusion of heterogeneous endpoints, including EOCRC incidence, advanced neoplasia, prediagnostic symptoms, screening yield, genetic susceptibility, and selected clinical outcomes, may limit direct comparability across domains. Although literature identification and thematic allocation followed a structured process documented in Supplementary Figure S1 and Supplementary Table S1, this work was designed as a narrative rather than a systematic review. The supplementary identification procedures were iterative and were not separately quantified. No formal risk-of-bias assessment, certainty-of-evidence grading, or de novo quantitative pooling was performed, and relevant studies not captured by the core search terminology or supplementary procedures may have been missed. The underlying evidence remains heterogeneous in EOCRC definitions, exposure and predictor ascertainment, covariate adjustment, and outcome definitions, limiting direct comparability across studies. Evidence across several domains remains uneven, and null or inconsistent findings may reflect limited power, misclassification, residual confounding, differences in healthcare access, or population-specific contexts rather than a true absence of association. This review was designed as a companion narrative synthesis to a separate review of modifiable metabolic, lifestyle, inflammatory, pharmacological, and socioeconomic determinants of EOCRC. Although scope-based selection and independent drafting were used to minimize overlap, some conceptual intersections between clinical, familial, and modifiable determinants are unavoidable. The predominance of evidence from Western and other high-income healthcare systems may also limit the generalizability of the findings to LMIC settings, where differences in healthcare infrastructure, screening availability, diagnostic capacity, and access to genetic services may substantially influence EOCRC detection and risk assessment. Future studies should therefore prioritize validation in underrepresented populations and healthcare systems.

The absence of a formal risk-of-bias assessment also limits the strength of interpretation of individual associations, particularly given the substantial contribution of observational evidence. Accordingly, findings should be regarded as a structured narrative synthesis rather than estimates of causal effects.

## 5. Conclusions

EOCRC differs from late-onset CRC across several clinically relevant dimensions. Younger patients more often present with distal or rectal tumors and symptom-driven diagnostic pathways and may have a higher likelihood of advanced-stage disease at diagnosis, highlighting the limitations of rigid age-based screening thresholds and the importance of timely evaluation of red-flag symptoms. Within the <50-year age range, risk increases progressively with age, whereas sex provides weaker discrimination and race or ethnicity should be interpreted as context-dependent information rather than as a deterministic biological marker. Family history remains one of the strongest risk markers, particularly when relatives are affected at younger ages, multiple relatives are affected, or advanced colorectal polyps are present. However, many EOCRC cases occur without a documented family history, supporting the need for broader risk assessment. Inherited susceptibility is heterogeneous: germline pathogenic variants meaningfully contribute to risk, while polygenic risk may offer additional research-oriented stratification but is not yet established for routine clinical use. Overall, effective EOCRC prevention and early detection will likely require integrated approaches combining symptoms, age, tumor distribution, familial and genetic risk, and locally appropriate screening strategies.

## Figures and Tables

**Table 1:** Clinical, demographic, familial, and inherited-susceptibility markers relevant to risk-stratified detection of early-onset colorectal cancer.

Domain	Marker or Clinical Feature	Study Characteristics	Role in the EOCRC Pathway	EOCRC Specificity	Main Evidence or Key Finding	Clinical Relevance for EOCRC Detection or Prevention	Refs.
Anatomical distribution	Distal or rectal tumor location.	Retrospective population-based study; *n* = 24,912; Canada	Clinical phenotype	EOCRC-enriched	EOCRC is more frequently diagnosed in the distal colon and rectum than late-onset CRC. In a population-based cohort, distal location was associated with diagnosis before age 50 years (OR 1.66, 95% CI 1.50–1.84).	A clinical-anatomical feature of EOCRC heterogeneity rather than an independent causal risk factor; may inform interpretation of symptom profiles and diagnostic trajectories.	[25]
Screening and diagnostic pathways	Screening colonoscopy in average-risk adults aged 45–49 years.	Systematic review and meta-analysis; 17 studies, *n* = 51,811; 4 continents	Detection	EOCRC-enriched	In a meta-analysis of asymptomatic average-risk individuals younger than 50 years, advanced colorectal neoplasia was detected in 3.6% of adults aged 45–49 years and 4.2% of those aged 50–59 years, indicating a clinically relevant, albeit somewhat lower, yield.	Supports current recommendations to consider average-risk screening starting at age 45 years in settings where such strategies are implemented; evidence reflects diagnostic yield and does not directly demonstrate reductions in EOCRC incidence or mortality.	[26]
Prediagnostic clinical features	Red-flag symptoms, anemia-related findings, and repeated healthcare encounters.	Fritz: Matched case–control study; *n* = 5075 EOCRC cases; USA Farooq: Population-based case–control study; *n* = 2567 CRC cases + 25,455 controls; Canada Demb: Systematic review and meta-analysis; 81 studies, *n* = 24,908,126; multiple international settings Sun: Population-based case–control study using EHR data; *n* = 1918 EOCRC cases + 9590 matched controls; USA Urback: Retrospective case–control study; *n* = 85 EOCRC cases + 80,211 controls; USA	Detection/diagnostic pathway	EOCRC-enriched	Rectal bleeding, abdominal or rectal pain, altered bowel habits, iron-deficiency anemia, gastrointestinal bleeding, and repeated healthcare encounters frequently precede EOCRC diagnosis. Low ferritin may provide an additional diagnostic clue, although evidence remains retrospective.	Should trigger timely diagnostic escalation and consideration of colonoscopy irrespective of young age.	[14,27,28,29,30]
Demographic marker	Increasing age within the <50-year range.	Low: Case–control study; *n* = 651 EOCRC cases + 67,416 controls; USA Bolwell: Retrospective colonoscopy-based case–control study; *n* = 13,006; USA Imperiale: Matched case–control study; *n* = 600 EOCRC cases + 2400 controls; USA	Development risk/risk stratification	Shared CRC risk factor	EOCRC risk appears to increase progressively with age within the <50-year range. Individual studies have reported approximately 5–8% higher odds of EOCRC per additional year of age.	Supports age-stratified risk assessment and heightened vigilance as individuals approach conventional screening age.	[31,32,33]
Demographic marker	Male sex.	Gu: Meta-analysis; 18 studies; 32,843 EOCRC cases; international O’Sullivan: Systematic review and meta-analysis; 20 studies; 47,692 EOCRC cases; North America, Europe, Asia, and Australia Ye: Meta-analysis; 28 studies; advanced colorectal neoplasia; geography not reported	Development risk/risk stratification	Shared CRC risk factor	Several studies have reported a modestly higher risk of EOCRC among men, although the magnitude of the association varied across populations.	Best used as one component of multivariable risk models rather than as an independent selection criterion.	[34,35,36]
Demographic and healthcare context	Race/ethnicity.	Gu: Meta-analysis; 18 studies; 32,843 EOCRC cases; international O’Sullivan: Systematic review and meta-analysis; 20 studies; 47,692 EOCRC cases; North America, Europe, Asia, and Australia Abu-Freha: Retrospective population-based cohort study; *n* = 61,679 CRC patients; Israel Tang: Global burden analysis using GBD 2019 data; global population; ages 40–49 years	Contextual/healthcare-related; risk stratification	Context-dependent/uncertain	Associations are heterogeneous across populations and may reflect differences in healthcare access, population structure, environmental exposures, and diagnostic pathways.	Interpret within local sociodemographic and healthcare context; race/ethnicity should not be treated as a deterministic biological marker.	[34,35,37,38]
Family history	First-degree relative with colorectal cancer.	Zhang: Systematic review and meta-analysis; 61 studies; observational evidence; UK Biobank genetic analysis; multiple populations O’Sullivan: Systematic review and meta-analysis; 20 studies, 47,692 EOCRC cases; North America, Europe, Asia, and Australia Roos: Systematic review and meta-analysis; 62 studies (42 case–control, 20 cohort); multiple populations Rosato: Pooled analysis of 3 case–control studies; *n* = 1690 (329 cases + 1361 controls); Italy and Switzerland	Development risk/risk stratification	EOCRC-enriched	Family history of CRC is among the most consistent EOCRC markers, although effect estimates vary according to study design, degree of relationship, age at diagnosis, and number of affected relatives.	Record degree of relationship, number of affected relatives, and age at diagnosis; use this information to guide earlier risk assessment and genetic referral.	[17,35,39,40]
Familial polyps	Advanced colorectal polyps or polyps diagnosed at a young age in relatives.	Song: Population-based case–control study; *n* = 401,813 (68,060 cases + 333,753 controls); Sweden	Development risk/risk stratification	Context-dependent/uncertain	A family history of colorectal polyps, particularly advanced adenomas, multiple lesions, or a younger age at diagnosis, is associated with increased CRC risk and exhibits dose–response patterns.	Include advanced or early-onset polyps in family-history assessment; EOCRC-specific implications require further validation.	[19]
Inherited susceptibility	Pathogenic germline variants in DNA mismatch repair genes, polyposis-associated genes, and other cancer-predisposition genes.	Vande Perre: Molecular comparative study; *n* = 87 EOCRC cases and 82 late-onset CRC comparators; France Yildiz: Hospital-based genetic study; *n* = 107 CRC patients; Cape Town and Johannesburg, South Africa Voer: Whole-exome sequencing discovery cohort (*n* = 55) + targeted resequencing replication cohort (*n* = 174); Netherlands and Germany Yurgelun: Multicenter cross-sectional cohort; *n* = 457; USA, Canada, Australia, and New Zealand; population-based and clinic-based recruitment	Development risk/risk stratification	EOCRC-enriched	Pathogenic germline variants are enriched in EOCRC, including apparently sporadic cases, and involve DNA mismatch repair genes, polyposis-associated genes, and other cancer-predisposition genes.	Provides a rationale for current consensus recommendations to perform germline testing in all patients with EOCRC, ideally before surgery, along with genetic counseling and appropriate cascade testing and surveillance for at-risk relatives.	[20,21,41,42]
Polygenic susceptibility	Polygenic risk score.	Archambault: Large multicohort genetic case–control study with independent prospective replication; discovery *n* = 108,062 (50,023 CRC cases + 58,039 controls), including 5479 EOCRC cases; replication *n* = 72,573; genetically defined European ancestry Chen: Population-based case–control study (DACHS), southwest Germany; 5104 CRC cases and 4131 controls, including 571 EOCRC cases (<55 years) and 417 younger controls	Development risk/risk stratification	EOCRC-enriched	Higher PRS categories are associated with substantially higher EOCRC risk, with some evidence suggesting stronger relative effects than in late-onset CRC; however, the clinical utility and generalizability of PRS remain unestablished.	A promising research tool that may eventually complement family history and monogenic testing, but is not yet established for routine clinical use and requires ancestry-aware validation and prospective implementation studies.	[22,43]

Abbreviations: CRC, colorectal cancer; EOCRC, early-onset colorectal cancer; OR, odds ratio; PRS, polygenic risk score. Refs., References.

## Data Availability

No new data were created or analyzed in this study. Data sharing is not applicable to this article.

## Supplementary Materials

The following supporting information can be downloaded at https://www.mdpi.com/article/10.3390/cancers18172877/s1, Figure S1: Flow diagram of literature identification, screening, eligibility assessment, and inclusion in the narrative synthesis; Table S1: Cross-reference and thematic classification of journal publications cited in the two companion narrative reviews.
