# Supplementary material for: Closing the Diagnostic Gap in Early-Onset Colorectal Cancer: Red-Flag Symptoms, Screening, and Inherited Risk
Source: Cancers (Basel). 2026 Sep 5;18(17):2877. doi: 10.3390/cancers18172877 (PMC13564552; doi:10.3390/cancers18172877)
Supplement: Supplementary file 1 [file cancers-18-02877-s001.zip › Supplementary Table S1. Cross-reference and thematic classification of journal publications cited in the two companion narrative reviews.pdf]

cancers

MDPI

Supplementary Table S1. Cross-reference and thematic classification of journal publications cited in the two companion narrative reviews.

Paper	*Cited in both companion reviews*	*Cited only in the life-course determinants and primary-prevention review*	*Cited only in the clinical presentation, diagnostic pathways, and inherited-susceptibility review*	*Topic*
Abdel-Rahman et al., 2022 - Patterns of colorectal cancer diagnosis among younger adults in a real-world, population-based cohort [24]	✓			Clinical/anatomical phenotype
Abu-Freha et al., 2025 - Early onset colorectal cancer, not just the age: Data from a large health organization [36]	✓			Clinical/anatomical phenotype
Akimoto et al., 2021 - Rising incidence of early-onset colorectal cancer - a call to action [50]	✓			Overview and emerging mechanisms
Alshenaifi et al., 2025 - Mutational and co-mutational landscape of early onset colorectal cancer [56]	✓			Molecular and tumor biology
Berrington de Gonzalez et al., 2025 - Trends in cancer incidence in younger and older adults: an international comparative analysis [9]	✓			Epidemiology and incidence trends
Bolwell et al., 2024 - Risk Factors Associated with Advanced Colorectal Neoplasia in Adults Younger than Age 45 [31]	✓			Mixed risk factors and prediction
Brenner et al., 2019 - National Trends in Colorectal Cancer Incidence Among Older and Younger Adults in Canada [72]	✓			Epidemiology and incidence trends
Cann et al., 2025 - Generations at Risk: Unveiling the Global Increase in Cancer at Both Ends of Adulthood [10]	✓			Overview and emerging mechanisms
Chang et al., 2021 - Risk factors for early-onset colorectal cancer: a population-based case–control study in Ontario, Canada [44]	✓			Mixed risk factors and prediction
Chen et al., 2022 - Alcohol consumption, polygenic risk score, and early- and late-onset colorectal cancer risk [42]	✓			Tobacco and alcohol

Paper	*Cited in both companion reviews*	*Cited only in the life-course determinants and primary-prevention review*	*Cited only in the clinical presentation, diagnostic pathways, and inherited-susceptibility review*	*Topic*
Chen et al., 2024 - Comparing cohort and period trends of early-onset colorectal cancer: a global analysis [23]	✓			Epidemiology and incidence trends
Danial et al., 2022 - Risk Factors of Young-Onset Colorectal Cancer: Analysis of a Large Population-Based Registry [51]	✓			Mixed risk factors and prediction
Deng et al., 2023 - Noninvasive predictive models based on lifestyle analysis and risk factors for early-onset colorectal cancer [55]	✓			Mixed risk factors and prediction
Erdem et al., 2025 - Snapshot cohort study on early-onset colorectal cancer in Northwestern Switzerland: are we doing enough or can we do more? [57]	✓			Clinical/anatomical phenotype
Exarchakou et al., 2019 - Colorectal cancer incidence among young adults in England: Trends by anatomical sub-site and deprivation [3]	✓			Socioeconomic/demographic disparities
Gu et al., 2022 - A risk scoring system to predict the individual incidence of early-onset colorectal cancer [33]	✓			Mixed risk factors and prediction
Hayes, 2021 - Advances in Understanding Early-Onset Colorectal Cancer [1]	✓			Overview and emerging mechanisms
Imperiale et al., 2023 - Risk Factors for Early-onset Sporadic Colorectal Cancer in Male Veterans [32]	✓			Mixed risk factors and prediction
Khan et al., 2015 - Dietary Practices, Addictive Behavior and Bowel Habits and Risk of Early Onset Colorectal Cancer: a Case Control Study [47]	✓			Diet and nutrition
Kim et al., 2016 - Different risk factors for advanced colorectal neoplasm in young adults [49]	✓			Mixed risk factors and prediction
Low et al., 2020 - Risk Factors for Early-Onset Colorectal Cancer [30]	✓			Mixed risk factors and prediction
O'Sullivan et al., 2022 - Risk Factors for Early-Onset Colorectal Cancer: A Systematic Review and Meta-analysis [34]	✓			Mixed risk factors and prediction

https://doi.org/10.3390/cancers18172877

Paper	*Cited in both companion reviews*	*Cited only in the life-course determinants and primary-prevention review*	*Cited only in the clinical presentation, diagnostic pathways, and inherited-susceptibility review*	*Topic*
O'Sullivan et al., 2024 - Risk factors for early-onset colorectal cancer: A Canadian prospective cohort study [53]	✓			Mixed risk factors and prediction
Pan et al., 2023 - Risk factors for early-onset colorectal cancer: A large-scale Chinese cohort study [54]	✓			Mixed risk factors and prediction
Park et al., 2022 - Clinical features of young onset colorectal cancer patients from a large cohort at a single cancer center [59]	✓			Clinical/anatomical phenotype
Rosato et al., 2013 - Risk factors for young-onset colorectal cancer [39]	✓			Mixed risk factors and prediction
Ruiz-Grajales et al., 2024 - Five-year overall survival of early- and late-onset colorectal cancer in Medellín, Colombia: a comparative study [73]	✓			Prognosis/surveillance/management
Sandhu et al., 2025 - Evaluating Risk Factors for Early-Onset Colorectal Cancer in a Large, Prospective Cohort [45]	✓			Mixed risk factors and prediction
Saraiva et al., 2023 - Early-onset colorectal cancer: A review of current knowledge [75]	✓			Overview and emerging mechanisms
Shen et al., 2021 - Risk Factors Associated With Early-Onset Colorectal Neoplasm in Chinese Youth: A Prospective Population-Based Study [52]	✓			Mixed risk factors and prediction
Siegel et al., 2023 - Colorectal cancer statistics, 2023 [2]	✓			Epidemiology and incidence trends
Silva et al., 2019 - Young-age onset colorectal cancer in Brazil: Analysis of incidence, clinical features, and outcomes in a tertiary cancer center [43]	✓			Epidemiology and incidence trends
Sun et al., 2025 - Predicting Early-Onset Colorectal Cancer in Individuals Below Screening Age Using Machine Learning and Real-World Data: Case Control Study [28]	✓			Mixed risk factors and prediction
Syed et al., 2019 - Old vs new: Risk factors predicting early onset colorectal cancer [46]	✓			Mixed risk factors and prediction

Paper	*Cited in both companion reviews*	*Cited only in the life-course determinants and primary-prevention review*	*Cited only in the clinical presentation, diagnostic pathways, and inherited-susceptibility review*	*Topic*
Tait et al., 2023 - Early-Onset Colorectal Cancer: Prevalence, Risk Factors, and Clinical Features Among Commercially Insured Adults in the United States [18]	✓			Clinical/anatomical phenotype
Tang et al., 2023 - Global burden of early-onset colorectal cancer among people aged 40-49 years from 1990 to 2019 and predictions to 2030 [48]	✓			Epidemiology and incidence trends
Tang et al., 2024 - Molecular characteristics of early-onset compared with late-onset colorectal cancer: a case controlled study [37]	✓			Molecular and tumor biology
The Lancet, 2026 - Cancer in young adults: out of the shadows? [12]	✓			Overview and emerging mechanisms
Tian et al., 2026 - Biological aging and generational shifts in early-onset cancer risk [11]	✓			Overview and emerging mechanisms
Urback et al., 2024 - Serum ferritin and the risk of early-onset colorectal cancer [29]	✓			Symptoms and diagnostic pathways
Willauer et al., 2019 - Clinical and molecular characterization of early-onset colorectal cancer [16]	✓			Molecular and tumor biology
Ye et al., 2023 - Risk factors for advanced colorectal neoplasm in young adults: a meta-analysis [35]	✓			Mixed risk factors and prediction
Zhang et al., 2023 - Field Synopsis of Environmental and Genetic Risk Factors of Sporadic Early-Onset Colorectal Cancer and Advanced Adenoma [17]	✓			Mixed risk factors and prediction
Albreht, 2021 - Europe's beating cancer plan-a new step towards more comprehensive and equitable cancer control in Europe		✓		Policy and primary prevention
Archambault et al., 2021 - Nongenetic Determinants of Risk for Early-Onset Colorectal Cancer		✓		Mixed risk factors and prediction
Ben-Aharon et al., 2023 - Pharmaceutical Agents as Potential Drivers in the Development of Early-Onset Colorectal Cancer: Case-Control Study		✓		Medications and antibiotics

Paper	*Cited in both companion reviews*	*Cited only in the life-course determinants and primary-prevention review*	*Cited only in the clinical presentation, diagnostic pathways, and inherited-susceptibility review*	*Topic*
Carroll et al., 2022 - Diet as a Risk Factor for Early-Onset Colorectal Adenoma and Carcinoma: A Systematic Review		✓		Diet and nutrition
Chang et al., 2024 - The Persistence of Hypertriglyceridemia and the Risk of Early Onset Colorectal Cancer According to Tumor Subsites: A Nationwide Population-Based Study		✓		Cardiometabolic factors
Chen et al., 2021 - Metabolic syndrome, metabolic comorbid conditions and risk of early-onset colorectal cancer		✓		Cardiometabolic factors
Galicia Pacheco et al., 2024 - Socio-Economic Inequalities in Beliefs About Cancer and its Causes: Evidence From two Population Surveys		✓		Socioeconomic/demographic disparities
Gong et al., 2025 - Epidemiological trends of early-onset gastrointestinal cancers from 1990 to 2021 and predictions for 2036: analysis from the global burden of disease study 2021		✓		Epidemiology and incidence trends
Hidayat et al., 2018 - Body fatness at an early age and risk of colorectal cancer		✓		Adiposity and weight trajectories
Jin et al., 2022 - Association Between Metabolic Syndrome and the Risk of Colorectal Cancer Diagnosed Before Age 50 Years According to Tumor Location		✓		Cardiometabolic factors
Kane et al., 2024 - Oral Antibiotic Use in Adulthood and Risk of Early-Onset Colorectal Cancer: A Case-Control Study		✓		Medications and antibiotics
Khoa Ta et al., 2023 - Association of diabetes mellitus with early-onset colorectal cancer: A systematic review and meta-analysis of 19 studies including 10 million individuals and 30,000 events		✓		Cardiometabolic factors
Kim et al., 2025 - Association Between Nonalcoholic Fatty Liver Disease and Risk of Early-onset Colorectal Cancer		✓		Cardiometabolic factors

https://doi.org/10.3390/cancers18172877

Paper	*Cited in both companion reviews*	*Cited only in the life-course determinants and primary-prevention review*	*Cited only in the clinical presentation, diagnostic pathways, and inherited-susceptibility review*	*Topic*
Li et al., 2021 - Association of Body Mass Index With Risk of Early-Onset Colorectal Cancer: Systematic Review and Meta-Analysis		✓		Adiposity and weight trajectories
Li et al., 2022 - Associations of Body Mass Index at Different Ages With Early-Onset Colorectal Cancer		✓		Adiposity and weight trajectories
Li et al., 2022 - Type 2 Diabetes and Risk of Early-Onset Colorectal Cancer		✓		Cardiometabolic factors
Li et al., 2023 - Associations of smoking with early- and late-onset colorectal cancer		✓		Tobacco and alcohol
Liu et al., 2019 - Association of Obesity With Risk of Early-Onset Colorectal Cancer Among Women		✓		Adiposity and weight trajectories
Lundqvist et al., 2023 - Autoimmune and Metabolic Diseases and the Risk of Early-Onset Colorectal Cancer, a Nationwide Nested Case-Control Study		✓		Inflammatory and clinical conditions
Luo et al., 2024 - Associations between blood glucose and early- and late-onset colorectal cancer: evidence from two prospective cohorts and Mendelian randomization analyses		✓		Cardiometabolic factors
McDowell et al., 2022 - Oral antibiotic use and early-onset colorectal cancer: findings from a case-control study using a national clinical database		✓		Medications and antibiotics
Nguyen et al., 2021 - The Sulfur Microbial Diet Is Associated With Increased Risk of Early-Onset Colorectal Cancer Precursors		✓		Diet and nutrition
Nguyen et al., 2022 - Antibiotic Therapy and Risk of Early-Onset Colorectal Cancer: A National Case-Control Study		✓		Medications and antibiotics
Ni et al., 2023 - Elucidating the Drivers for the Rising Incidence of Early-Onset Colorectal Cancer: How Ecologic Studies Could Help and What Is Next		✓		Overview and emerging mechanisms
Norat et al., 2015 - European code against cancer 4th edition: diet and cancer		✓		Policy and primary prevention

Paper	*Cited in both companion reviews*	*Cited only in the life-course determinants and primary-prevention review*	*Cited only in the clinical presentation, diagnostic pathways, and inherited-susceptibility review*	*Topic*
Puzzono et al., 2022 - Diet and Lifestyle Habits in Early-Onset Colorectal Cancer: A Pilot Case-Control Study		✓		Diet and nutrition
Schebesta et al., 2020 - The European union farm to fork strategy		✓		Policy and primary prevention
Schumacher et al., 2021 - Metabolic Risk Factors Associated with Early-Onset Colorectal Adenocarcinoma: A Case-Control Study at Kaiser Permanente Southern California		✓		Cardiometabolic factors
Song et al., 2023 - Association of changes in obesity and abdominal obesity status with early-onset colorectal cancer risk: a nationwide population-based cohort study		✓		Adiposity and weight trajectories
Wang et al., 2022 - Time Trend and Association of Early-Onset Colorectal Cancer with Diverticular Disease in the United States: 2010-2021		✓		Inflammatory and clinical conditions
Wang et al., 2025 - Global Epidemiology of Early-Onset Digestive System Malignancy: A Systematic Analysis for the Global Burden of Disease Study 2021		✓		Epidemiology and incidence trends
Zhang et al., 2023 - Knowledge mapping of early-onset colorectal cancer from 2000 to 2022: A bibliometric analysis		✓		Overview and emerging mechanisms
Alsiary et al., 2023 - Early-Onset Colorectal Cancer Survival among Unscreened Population - Multicenter Cohort Retrospective Analysis [69]			✓	Prognosis/surveillance/management
Archambault et al., 2020 - Cumulative Burden of Colorectal Cancer–Associated Genetic Variants Is More Strongly Associated With Early-Onset vs Late-Onset Cancer [22]			✓	Hereditary and genetic susceptibility
Baronas et al., 2024 - Symptom Burden and Time from Symptom Onset to Cancer Diagnosis in Patients with Early-Onset Colorectal Cancer: A Multicenter Retrospective Analysis [13]			✓	Symptoms and diagnostic pathways

Paper	*Cited in both companion reviews*	*Cited only in the life-course determinants and primary-prevention review*	*Cited only in the clinical presentation, diagnostic pathways, and inherited-susceptibility review*	*Topic*
Barot et al., 2025 - Incidence trends and long-term survival in early-onset colorectal cancer: a nationwide Swedish study [74]			✓	Epidemiology and incidence trends
Brim et al., 2025 - Trends and Symptoms Among Increasing Proportion of African Americans with Early-Onset Colorectal Cancer over a 60-Year Period [7]			✓	Socioeconomic/demographic disparities
Cavestro Giulia et al., 2022 - Delphi initiative for early-onset colorectal cancer (DIRECt). International Management Guidelines [66]			✓	Hereditary and genetic susceptibility
Chen et al., 2022 - Early-Onset Colorectal Cancer Is Associated with a Lower Risk of Metachronous Advanced Neoplasia than Traditional-Onset Colorectal Cancer [79]			✓	Prognosis/surveillance/management
Chen et al., 2025 - Prognosis and clinicopathological features of patients with early-onset and late-onset colorectal cancer with second primary malignancies [80]			✓	Prognosis/surveillance/management
Cheng et al., 2021 - Analysis of Survival Among Adults With Early-Onset Colorectal Cancer in the National Cancer Database [67]			✓	Prognosis/surveillance/management
de Voer et al., 2016 - Identification of Novel Candidate Genes for Early-Onset Colorectal Cancer Susceptibility [40]			✓	Hereditary and genetic susceptibility
Demb et al., 2024 - Red Flag Signs and Symptoms for Patients With Early-Onset Colorectal Cancer: A Systematic Review and Meta-Analysis [27]			✓	Symptoms and diagnostic pathways
Farooq et al., 2022 - Patterns of healthcare utilization leading to diagnosis of young-onset colorectal cancer (yCRC): population-based case-control study [26]			✓	Symptoms and diagnostic pathways
Fritz et al., 2023 - Red-flag signs and symptoms for earlier diagnosis of early-onset colorectal cancer [14]			✓	Symptoms and diagnostic pathways

Paper	*Cited in both companion reviews*	*Cited only in the life-course determinants and primary-prevention review*	*Cited only in the clinical presentation, diagnostic pathways, and inherited-susceptibility review*	*Topic*
Giráldez et al., 2012 - Susceptibility genetic variants associated with early-onset colorectal cancer [65]			✓	Hereditary and genetic susceptibility
Golubicki et al., 2020 - Germline biallelic Mcm8 variants are associated with early-onset Lynch-like syndrome [62]			✓	Hereditary and genetic susceptibility
Iberri Jaime et al., 2025 - Early-Onset Colorectal Cancer: A New Clinical Profile in Patients Treated at a Referral Hospital in Mexico City [70]			✓	Clinical/anatomical phenotype
Imperiale et al., 2025 - A qualitative study of a new metric for estimating early-onset colorectal cancer risk in male veterans: 'Colon Age' [77]			✓	Mixed risk factors and prediction
Kędzia-Berut et al., 2023 - Colorectal Cancer: Is it Still a Disease of the Elderly? [71]			✓	Overview and emerging mechanisms
Kolarich et al., 2018 - Rectal cancer patients younger than 50 years lack a survival benefit from NCCN guideline-directed treatment for stage II and III disease [82]			✓	Prognosis/surveillance/management
Kolb et al., 2021 - Early-Age Onset Colorectal Neoplasia in Average-Risk Individuals Undergoing Screening Colonoscopy: A Systematic Review and Meta-Analysis [25]			✓	Screening and early detection
Laskar et al., 2024 - Genome-wide association studies and Mendelian randomization analyses provide insights into the causes of early-onset colorectal cancer [64]			✓	Hereditary and genetic susceptibility
Lin et al., 2025 - Early- vs. late-onset colon cancer: clinicopathological insights and survival outcomes in an East Asian cohort [68]			✓	Prognosis/surveillance/management
Malik et al., 2018 - Metachronous colorectal cancer following segmental or extended colectomy in Lynch syndrome: a systematic review and meta-analysis [60]			✓	Hereditary and genetic susceptibility

https://doi.org/10.3390/cancers18172877

Paper	*Cited in both companion reviews*	*Cited only in the life-course determinants and primary-prevention review*	*Cited only in the clinical presentation, diagnostic pathways, and inherited-susceptibility review*	*Topic*
Monge et al., 2024 - WNT and TGF-Beta Pathway Alterations in Early-Onset Colorectal Cancer Among Hispanic/Latino Populations [6]			✓	Molecular and tumor biology
Monge et al., 2025 - Molecular Heterogeneity in Early-Onset Colorectal Cancer: Pathway-Specific Insights in High-Risk Populations [8]			✓	Molecular and tumor biology
Mueller et al., 2021 - Colorectal cancer of the young displays distinct features of aggressive tumor biology: A single-center cohort study [15]			✓	Molecular and tumor biology
Quach et al., 2012 - Clinical, endoscopic and pathogical characteristics of early-onset colorectal cancer in Vietnamese [58]			✓	Clinical/anatomical phenotype
Roos et al., 2019 - Effects of Family History on Relative and Absolute Risks for Colorectal Cancer: A Systematic Review and Meta-Analysis [38]			✓	Family history and familial polyps
Salem et al., 2020 - Molecular Analyses of Left- and Right-Sided Tumors in Adolescents and Young Adults with Colorectal Cancer [76]			✓	Molecular and tumor biology
Song et al., 2021 - Risk of colorectal cancer in first degree relatives of patients with colorectal polyps: nationwide case-control study in Sweden [19]			✓	Family history and familial polyps
Tashkandi, 2025 - The Rise of Early-Onset Colorectal Cancer: Clinical Characteristics and Outcomes in a Young Patient Population [81]			✓	Clinical/anatomical phenotype
Torres et al., 2025 - Unraveling Early Onset Disparities and Determinants: An Analysis of Colorectal Cancer Outcomes and Trends in Texas [4]			✓	Socioeconomic/demographic disparities
van Roon et al., 2010 - Early onset MSI-H colon cancer with MLH1 promoter methylation, is there a genetic predisposition? [61]			✓	Hereditary and genetic susceptibility
Vande Perre et al., 2025 - Germline Sequencing of Familial and Sporadic Early-Onset Colorectal Cancer: A Novel Pattern of Genes [20]			✓	Hereditary and genetic susceptibility

Paper	*Cited in both companion reviews*	*Cited only in the life-course determinants and primary-prevention review*	*Cited only in the clinical presentation, diagnostic pathways, and inherited-susceptibility review*	*Topic*
Wang et al., 2022 - High risk and early onset of cancer in Chinese patients with Peutz-Jeghers syndrome [78]			✓	Hereditary and genetic susceptibility
Wang et al., 2023 - Identification of specific susceptibility loci for the early-onset colorectal cancer [63]			✓	Hereditary and genetic susceptibility
Yildiz et al., 2023 - Genetic insights: High germline variant rate in an indigenous African cohort with early-onset colorectal cancer [21]			✓	Hereditary and genetic susceptibility
Yurgelun et al., 2015 - Germline TP53 Mutations in Patients With Early-Onset Colorectal Cancer in the Colon Cancer Family Registry [41]			✓	Hereditary and genetic susceptibility
Zaki et al., 2023 - Racial and Ethnic Disparities in Early-Onset Colorectal Cancer Survival [5]			✓	Socioeconomic/demographic disparities

https://doi.org/10.3390/cancers18172877
